# A core-shell multifunctional microneedle patch accelerates infected diabetic wound healing

**DOI:** 10.1186/s13287-025-04738-z

**Published:** 2025-10-31

**Authors:** Kang Wei, Lu He, Yiran Shi, Wen Zhang, Meng Wu, Shengdan Zhang, Haijie Wang, Huanhuan Xu, Liangcong Hu, Wei Li, Liang Guo

**Affiliations:** 1 Department of Plastic Surgery, Zhongnan Hospital of Wuhan University, 169 East Lake Road, Wuchang District, Wuhan, 430071 China; 2 Department of Obstetrics and GynecologyWuhan Children’s HospitalTongji Medical College, Huazhong University of Science and Technology, 100 Xianggang Road, Wuhan, 430000 China; 3 TaiKang Center for Life and Medical Sciences, Wuhan University, Wuhan, 430071 P. R. China; 4 Division of Surgery and Interventional Science, Royal Free Hospital of University College London, Pond Street, London, NW3 2QG UK; 5 Department of Stomatology, Zhongnan Hospital of Wuhan University, 169 East Lake Road, Wuchang District, Wuhan, 430071 China

**Keywords:** Microneedles, Diabetic wound healing, Inflammation, Palmatine, SDFP

## Abstract

Diabetic wounds exhibit impaired angiogenesis, drug-resistant bacterial infections, and oxidative damage to the microenvironment, presenting ongoing clinical challenges. In this research, we designed a core-shell structured multifunctional microneedle (MN) array patch (CS-SDFP/HA-Ag@MOF-PAL) to expedite the healing of infected diabetic wounds. The core consists of 10,000 MV hyaluronic acid (HA) and sucrose, integrated with Ag@MOF loaded with Palmatine (Ag@MOF-PAL). The shell, composed of chitosan (CS), encapsulates SDFP (CS-SDFP). Upon application to an infected diabetic wound, the shell swells, releasing the HA core, which dissolves to release Ag@MOF-PAL. The Ag@MOF targets and eradicates bacteria and eliminates biofilms of bacteria in the wound area, such as *Staphylococcus aureus*, *Escherichia coli*, and *Pseudomonas aeruginosa*, while PAL mitigates chronic inflammation in wound macrophages via the NF-κB and MAPK signaling pathways. As the wound microenvironment normalizes, the SDFP released from the swollen CS recruits adipose-derived stem cells (ADSCs) and promotes angiogenesis to facilitate wound repair. Our experiments demonstrate that CS-SDFP/HA-Ag@MOF-PAL accelerates wound healing in a diabetic rat model with *Pseudomonas aeruginosa*-infected wounds. The property of this core-shell structured multifunctional MN patch presents considerable potential for the development of therapeutic strategies aimed at the healing of infected diabetic wounds.

**Supplementary Information:**

The online version contains supplementary material available at 10.1186/s13287-025-04738-z.

## Introduction

Diabetic complications represent a significant threat to patient health, leading to decreased quality of life, increased healthcare costs, and elevated mortality rates [1, 2]. The global prevalence of adult diabetes is projected to rise to 7.7% by 2030 [3]. Diabetic foot ulcers (DFUs), which are the most prevalent complication of diabetes, encompass suboptimal glycaemic control, peripheral neuropathy, peripheral vascular disease, and immunosuppression [4, 5], affecting 15–25% of individuals with diabetes mellitus [6]. It is estimated that approximately 60% of amputations are associated with DFU [7]. Standard DFU treatment involves debridement, anti-infective therapies, and wound dressing [8]. However, the effectiveness of these approaches is often suboptimal. The excessive use of antibiotics represents a primary contributor to the continuous increase of antibiotic resistance in bacteria [9]. Moreover, the need for frequent dressing replacements results in pain and additional trauma to the wound, markedly diminishing patient adherence [10]. Consequently, innovative and efficient approaches for accelerating wound recovery have become increasingly imperative.

Microneedle (MN), consisting of an array of needles mounted on a base, offer a promising solution by bypassing physical barriers to directly deliver therapeutic agents to deeper tissues, thus improving drug stability and delivery efficacy [11, 12]. MN represent a minimally invasive and highly effective transdermal drug delivery platform, frequently applied to promote wound healing [13]. The ability of MN tips to penetrate biofilms aids in the dispersion of antimicrobial agents into infected tissues, thereby enhancing their antimicrobial activity [14]. However, the degradation of bacteria and necrotic cells in the wound releases pathogenic nucleic acid fragments, triggering inflammatory responses that contribute to excessive cytokine secretion and sustained immune activation [15].

Nanoparticles can be delivered through MN to accelerate normal and diabetic wound healing [16–19]. The metal-organic frameworks (MOFs) is a unique class of nanoporous materials formed from metal ions interconnected by organic ligands via coordinate covalent bonds. Given their remarkable structural attributes, MOFs are emerging as prospective nanoplatforms for precise drug delivery [20, 21]. By harnessing the distinct properties of different MOFs materials, biomaterials functionalized with integrated MOFs can attain improved bone regeneration, antibacterial, anti-inflammatory, and anti-tumor capabilities [22]. Unlike natural bactericides, MOFs present multiple advantages, such as broad-spectrum antibacterial activity, high efficacy, extended duration of action, structural tunability, and thermal resilience [23]. Ag-MOFs (Ag@MOF), such as [Ag_5_(PYDC)_2_(OH)], have demonstrated potential as antimicrobial agents against bacteria, yeasts, and molds [24]. The Ag@MOF exhibits distinct rod-shaped structures, which facilitate the uniform distribution of metal within the polymer matrix while promoting the sustained release of Ag^+^ ions to prevent aggregation. Palmatine (PAL), an isoquinoline alkaloid classified under the protoberberine group, has demonstrated a range of properties, encompassing anti-inflammatory, antioxidant, neuroprotective, antibacterial, antiviral, and anticancer effects [25]. Stromal-derived factor-1α (SDF1α), a chemokine, plays a pivotal role in stem cell migration for tissue regeneration [26]. Nonetheless, the therapeutic use of SDF1α is hindered by issues related to its high production cost, storage instability, and transport limitations [27]. The SDF1α polypeptide (SDFP), a cost-effective alternative, shares the same receptor activation domain as the full-length protein and similarly promotes stem cell migration [28].

**Fig. 1:**
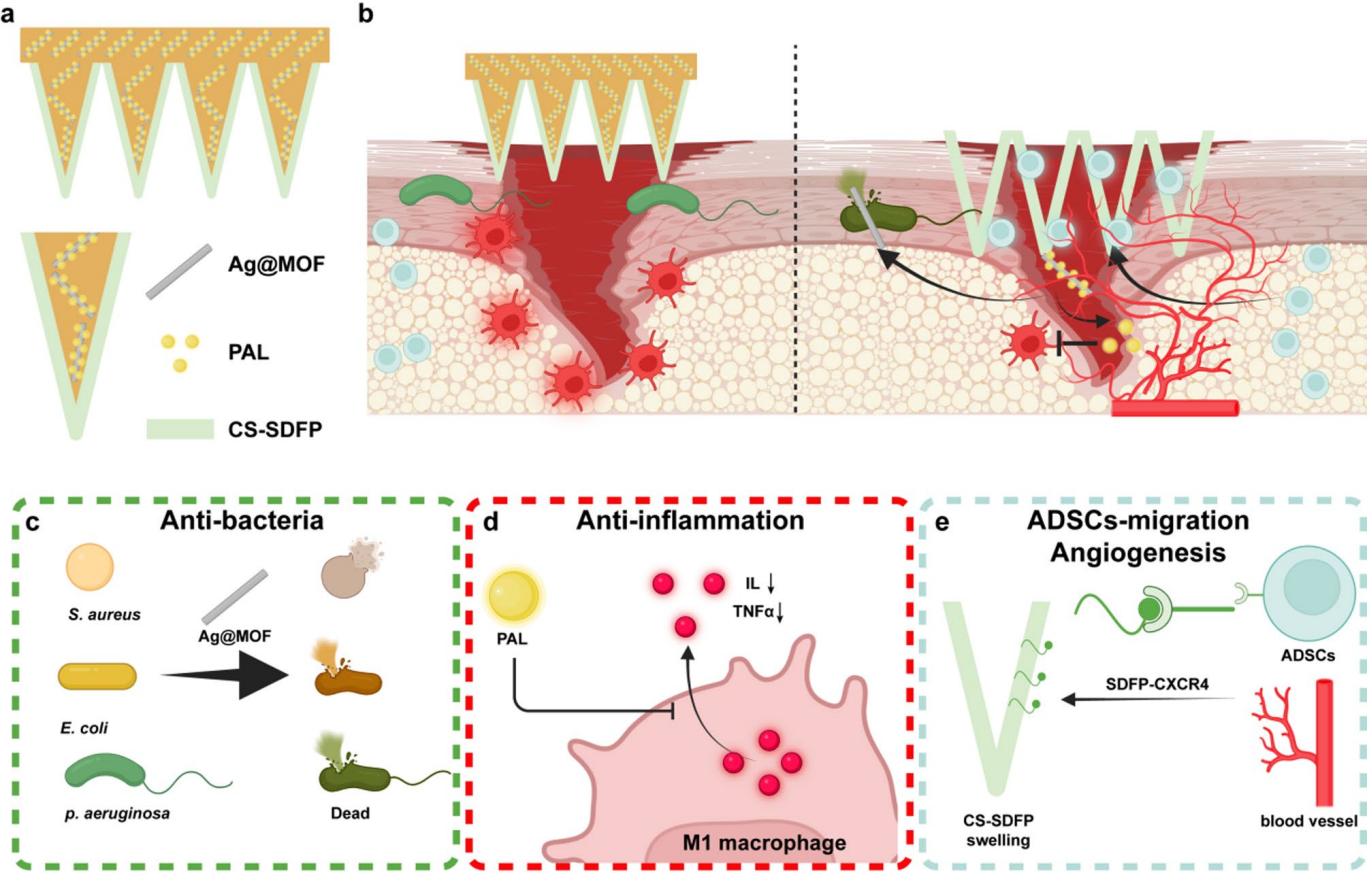
The mechanism diagram of CS-SDFP/HA-Ag@MOF-PAL MN patches promoting infected diabetic wound healing. **a** Schematic illustration of CS-SDFP/HA-Ag@MOF-PAL Core-Shell MN patches. **b** Working mechanism of CS-SDFP/HA-Ag@MOF-PAL MN patches. **c** The anti-bacteria function of Ag@MOF. **d** The anti-inflammation function of PAL. **e** The ADSCs-migration and angiogenesis function of SDFP

This study presents a core-shell structured MN patch designed to accelerate the healing of infected diabetic wounds (Fig. 1). The core comprises 10,000 MV hyaluronic acid (HA), sucrose, and Ag@MOF loaded with PAL (Ag@MOF-PAL), while the shell is composed of chitosan (CS), encapsulating SDFP (CS-SDFP). In the process of wound healing, CS assumes a pivotal role. It offers a matrix that facilitates three-dimensional tissue growth, stimulates tissue organization, and promotes cell proliferation. Moreover, CS can activate macrophages and boost their tumoricidal activity [29]. To achieve rapid healing of diabetic infected wounds, we designed a core-shell MN patch (CS-SDFP/HA-Ag@MOF-PAL MN patch). This core-shell MN patch is engineered to modulate bacterial infection, chronic inflammation, and stem cell migration. Upon application to the infected diabetic wound, the swelling shell adheres to the wound site, while the core gradually dissolves and releases Ag@MOF-PAL. First, Ag@MOF exhibits strong antibacterial activity at the wound site. PAL then effectively mitigates the chronic inflammatory response. Finally, once the wound microenvironment stabilizes, the swelling CS releases SDFP, which recruits stem cells from healthy tissue to promote wound healing. This innovative design accelerates the healing process of infected diabetic wounds in diabetic mouse models.

## Results

### Characterization and cytotoxicity of CS-SDFP/HA-Ag@MOF-PAL MN patches

**Fig. 2:**
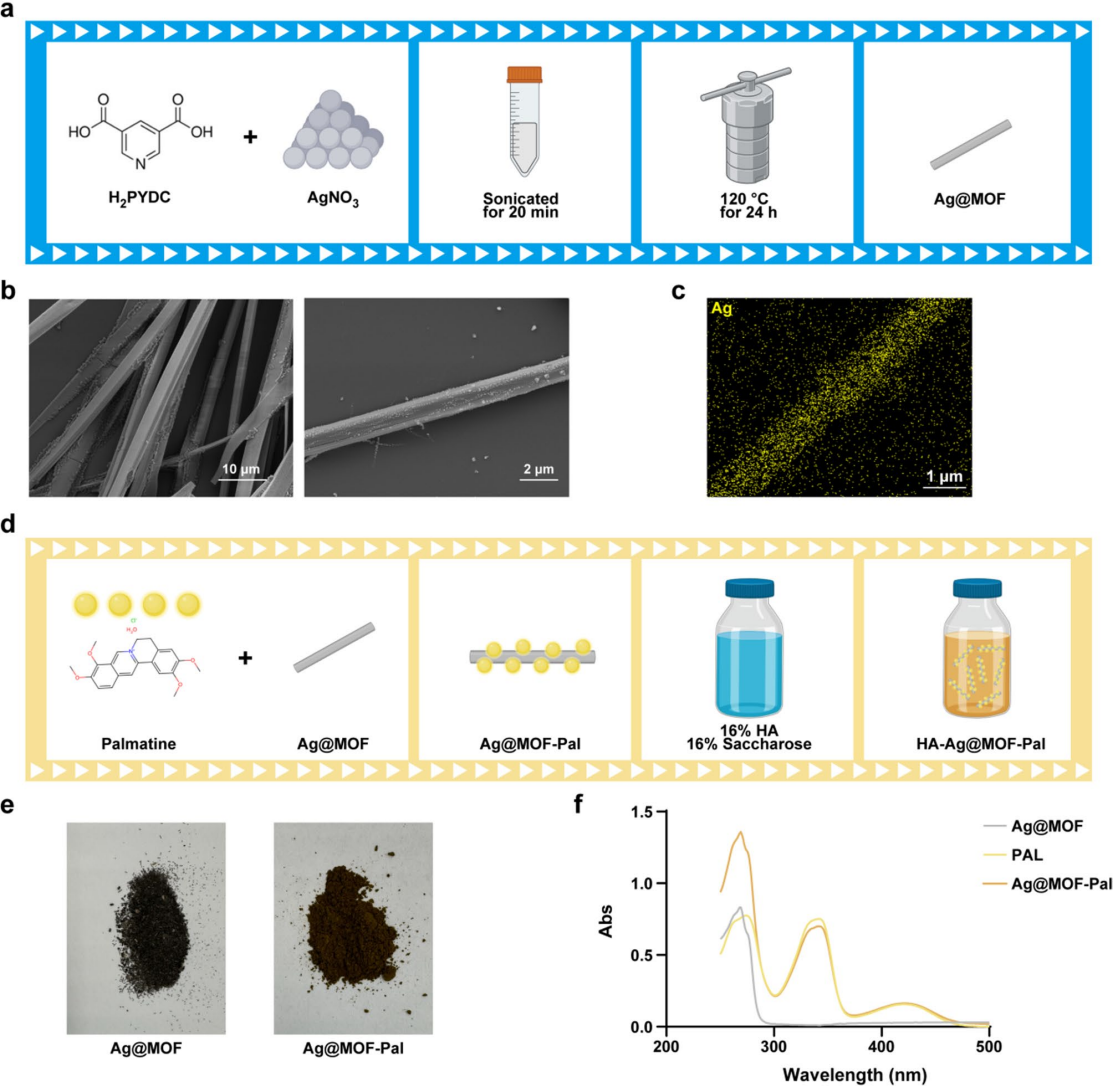
Ag@MOF-PAL synthesis. **a** Schematic illustration of Ag@MOF synthesis. **b** FESEM image of Ag@MOF. **c** The EDS elemental mapping analyses of Ag@MOF. **d** Schematic illustration of Ag@MOF-PAL synthesis. **e**The images of Ag@MOF and Ag@MOF-PAL. **f** UV-vis spectrum of Ag@MOF, PAL and Ag@MOF-PAL

The flowchart elucidates the composition process of Ag@MOF (Fig. 2a). The Scanning electron microscopy (SEM) images depicted the structure of synthesized Ag@MOF (Fig. 2b). Elemental mapping analysis confirms that the Ag@MOF is rich in Ag content (Fig. 2c). After synthesizing Ag@MOF with PAL to form Ag@MOF/PAL, the Ag@MOF/PAL mixture was combined with 16% HA and 16% sucrose to produce HA-Ag@MOF-PAL (Fig. 2d). Figure 2e displays images of Ag@MOF and Ag@MOF-PAL. The ultraviolet-visible spectra exhibited different synthetic samples (Fig. 2f).

**Fig. 3:**
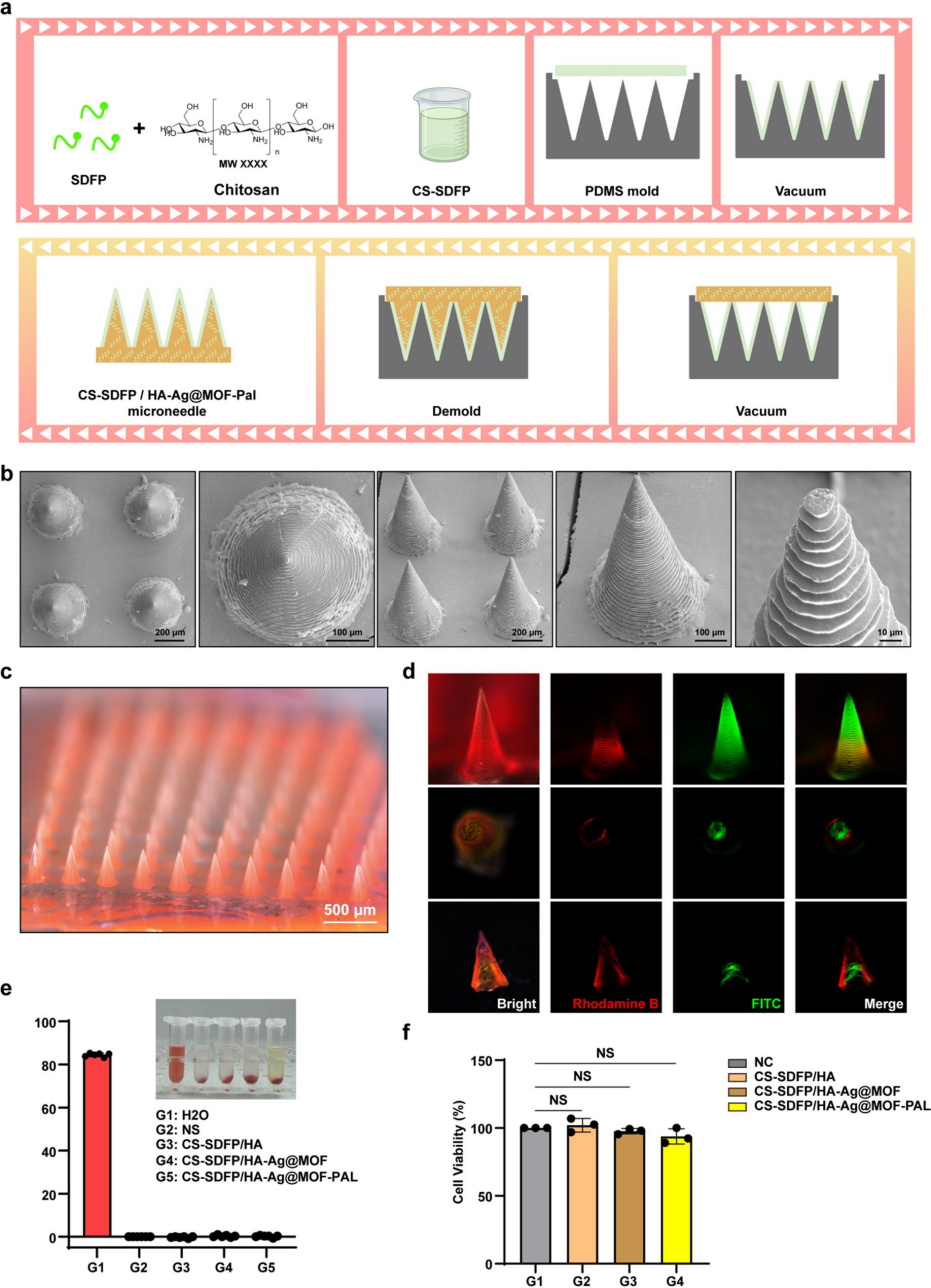
The fabrication and characterization of CS-SDFP/HA-Ag@MOF-PAL MN patches. **a** Schematic illustration of CS-SDFP/HA-Ag@MOF-PAL MN patches. **b** FESEM image of CS-SDFP/HA-Ag@MOF-PAL MN patches. **c** Representative bright-field microscopy images of CS-SDFP/HA-Ag@MOF-PAL MN patches. **d** Fluorescence image of core-shell structure of CS-SDFP/HA-Ag@MOF-PAL MN patches. **e** The hemolysis rate of red blood cells following a 6 h incubation with various MN patches at 37 °C. **f** Cell viability of HUVECs treated with different MN patches

Following the preparation of the SDFP and CS mixture, 100 µL of CS-SDFP was placed on the PMDS mold. After 30 min of vacuum-assisted micromolding, 100 µL of HA-Ag@MOF-PAL was added. After drying, CS-SDFP/HA-Ag@MOF-PAL MN patches were obtained (Fig. 3a). The FESEM showed that the CS-SDFP/HA-Ag@MOF-PAL MNs exhibited a conical morphology (Fig. 3b). To further illustrate the core-shell structure of the CS-SDFP/HA-Ag@MOF-PAL MN patches, the CS-SDFP was labeled with the fluorescent dye Rhodamine B, and the HA-Ag@MOF-PAL was labeled with FITC. The MNs were uniformly arranged along the substrate layer (Fig. 3c), and the core-shell structure was confirmed by cross-sectional imaging of the MN (Fig. 3d). The core-shell MN patch consisted of conical microneedles. Each microneedle had a base radius of 200 μm, a height of 850 μm, and a tip radius of approximately 10 μm. These microneedles were arrayed in a 10 × 10 pattern within an area measuring 7 mm × 7 mm. Force-displacement curves of the CS-SDFP/HA-Ag@MOF-PAL MN patches was shown in Fig. S1a. Additionally, incubation of red blood cells with the MN patch displayed no significant hemolysis (Fig. 3e). Furthermore, MN extract did not affect HUVECs activity, as demonstrated in the CCK8 cell viability assay (Fig. 3f). These results confirm that the CS-SDFP/HA-Ag@MOF-PAL MN patches possess satisfactory biosafety.

### The anti-bacterial effects of CS-SDFP/HA-Ag@MOF-PAL MN patches in vitro

**Fig. 4:**
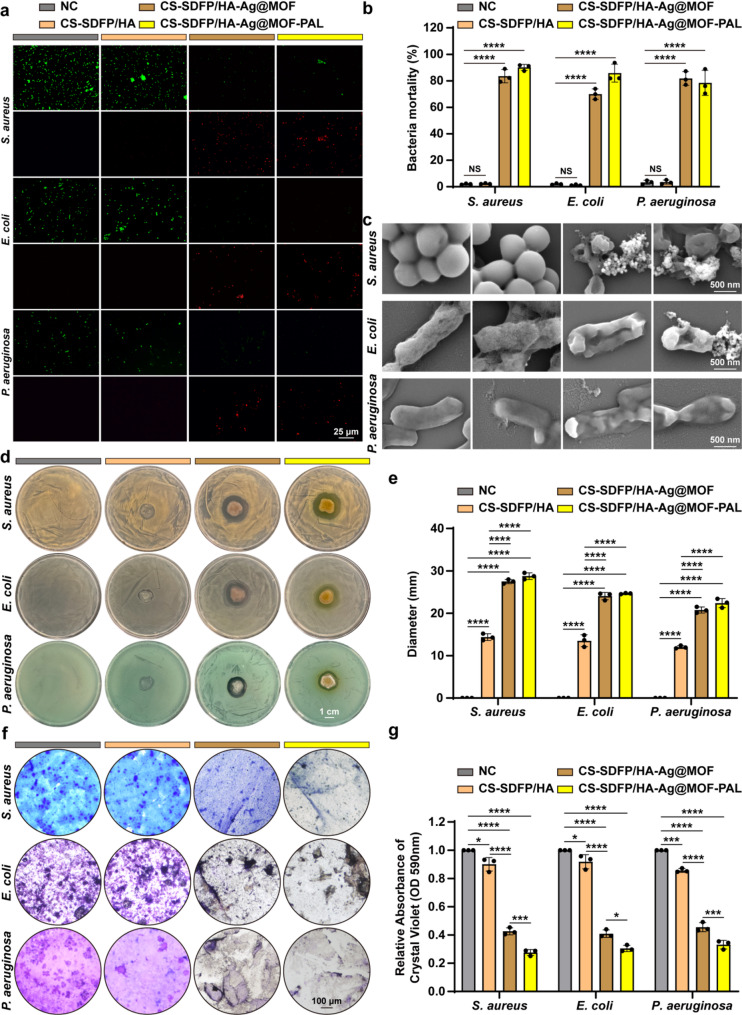
The anti-bacterial effects of CS-SDFP/HA-Ag@MOF-PAL MN patches in vitro. **a** Live/dead assay of *S. aureus*, *E. coli*, and *P. aeruginosa* after incubation with different MN patches. Scale bar = 25 μm. **b** Bacteria mortality of *S. aureus*, *E. coli*, and *P. aeruginosa* after being treated with different MN patches. **c** FESEM image of *S. aureus*, *E. coli*, and *P. aeruginosa* after being treated with different MN patches. Scale bar = 500 nm. **d** Representative bright-field images of inhibition zones against *S. aureus*, *E. coli*, and *P. aeruginosa* with different MN treatments for 12 h. Scale bar = 1 cm. **e** Diameter of the inhibition zones against *S. aureus*, *E. coli*, and *P. aeruginosa*. **f** Biofilms image of *S. aureus*, *E. coli*, and *P. aeruginosa* stained with crystal violet after being treated with different MNs. Scale bar = 100 μm. **g** Relative Absorbance of Crystal Violet (OD 590 nm) of *S. aureus*, *E. coli*, and *P. aeruginosa* biological film after being treated with different MNs in (**f**). Data are represented as mean +/− SD (*n* = 3). *p < 0.05, **p < 0.01, ***p < 0.001, ****p < 0.0001. The NS indicates no significance

Prevalent bacteria that cause wound infections include *Staphylococcus aureus* (*S. aureus*), *Escherichia coli* (*E. coli*), and *Pseudomonas aeruginosa* (*P. aeruginosa*) [30]. *P. aeruginosa* is the most common pathogen responsible for healthcare-associated infections [31, 32]. To evaluate the antibacterial properties of the CS-SDFP/HA-Ag@MOF-PAL MN patches, a bacterial viability staining assay utilizing the SYTO-9/PI method was conducted on *S. aureus*, *E. coli*, and *P. aeruginosa* following exposure to the MN patches for 24 h. The results showed that the viability of all three bacterial strains was significantly reduced after treatment with the MN patches (Fig. 4a, b). Further investigation of the bacterial cell membrane morphology was carried out using field emission SEM (FESEM) following the application of MN patches. In the negative control (NC) and CS-SDFP/HA MN patch groups, *S. aureus*, *E. coli*, and *P. aeruginosa* exhibited relatively smooth, spherical morphologies with intact cell membranes. In contrast, diminished membrane integrity was noted in the groups administered MN patches comprising Ag@MOF (Fig. 4c). Additionally, upon application of MN patches to cultures of *S. aureus*, *E. coli*, or *P. aeruginosa*, the CS-SDFP/HA-Ag@MOF MN patches produced inhibition zones comparable to those of the CS-SDFP/HA-Ag@MOF-PAL MN patches (Fig. 4d). The diameters of the antibacterial zone were measured as 28.77 ± 0.77 mm for *S. aureus*, 24.66 ± 0.12 mm for *E. coli*, and 22.41 ± 1.06 mm for *P. aeruginosa* (Fig. 4e). Moreover, the MN patch containing Ag@MOF was effective in eradicating biofilms of *S. aureus*, *E. coli*, and *P. aeruginosa* in vitro. These biofilms are known to contribute significantly to the development of drug resistance in pathogenic bacteria, as demonstrated by crystal violet staining (Fig. 4f-g). Therefore, the antibacterial activity of the MN patches was primarily attributed to the Ag@MOF component.

### CS-SDFP/HA-Ag@MOF-PAL MN patches accelerated infected diabetic wound healing in vivo

**Fig. 5:**
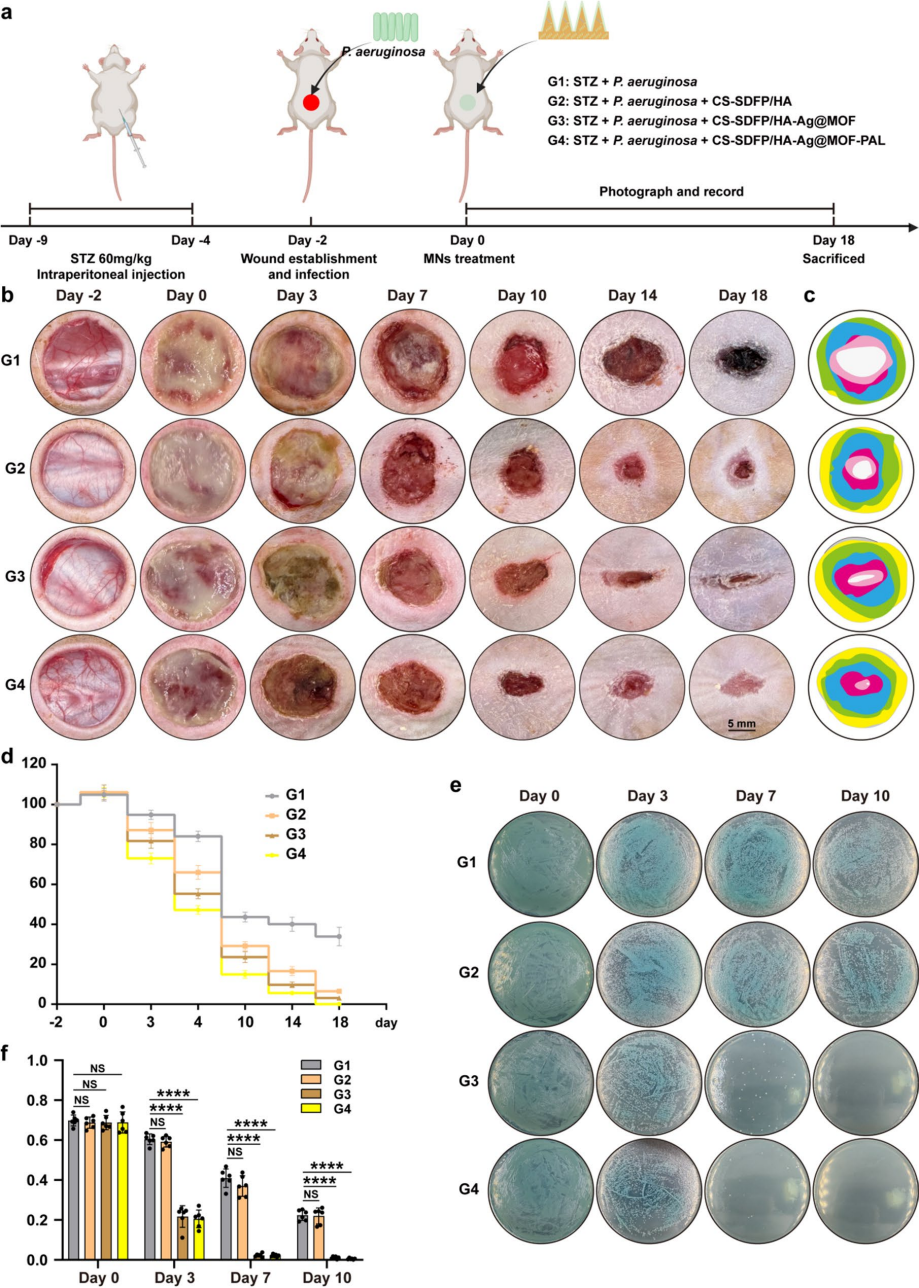
CS-SDFP/HA-Ag@MOF-PAL MN Patches Accelerated Infected Diabetic Wound Healing in vivo. **a** Time scheme of the experiment in vivo. **b** Representative pictures of the rat skin wounds on days − 2, 0, 3, 7, 10, 14, and 18 after receiving different treatments. Scale bar = 5 mm (**c**) Schematic diagram of wound morphological changes in different groups within 18 days. **d** Wound remaining ratio of the rat skin wounds with different treatments. **e** Representative image of *P. aeruginosa* that were separated from rat wound on 0, 3, 7, and 10 days in different groups. **f** The OD600 values of *P. aeruginosa* isolated from the wound in LB liquid medium for 24 h on 0, 3, 7, and 10 days in different groups. *p < 0.05, **p < 0.01, ***p < 0.001, ****p < 0.0001. The NS indicates no significance

A full-thickness skin excision model in rodents, encompassing mice, is widely acknowledged as a clinically pertinent method for assessing wound healing [33, 34]. In the present study, a full-thickness skin wound (diameter: 1.5 cm) infected with *P. aeruginosa* was created on the backs of diabetic Sprague Dawley rats. Following 2 days of *P. aeruginosa* infection, the wound was treated with MN patches (Fig. 5a). Following intraperitoneal injection of streptozotocin (STZ), the rats showed an increase in blood glucose levels (Fig. S1b). On days 0, 3, 7, 10, 14, and 18, wound images were captured for each group (Fig. 5b), and changes in wound size over time were evaluated utilizing a simulation method (Fig. 5c). Analysis of the wound healing rate demonstrated that treatment with CS-SDFP/HA-Ag@MOF-PAL MN patches significantly accelerated the healing of infected wounds in diabetic rats (Fig. 5d). No substantial toxicity was observed in the major organs of the rats (Fig. S1c). To assess the antibacterial effectiveness of CS-SDFP/HA-Ag@MOF-PAL MN patches, *P. aeruginosa* was procured from the wounds on days 0, 3, 7, 10, 14, and 18 and cultured on LB agar plates (Fig. 5e). The optical density at OD 600 nm was measured after culturing *P. aeruginosa* isolated from the wounds in LB liquid medium for 24 h (Fig. 5f), indicated near-total eradication of viable bacteria following treatment with CS-SDFP/HA-Ag@MOF-PAL MN patches. In comparison to other groups, no viable *P. aeruginosa* was detected in wounds treated with CS-SDFP/HA-Ag@MOF-PAL, highlighting the superior antimicrobial efficacy of the MN patches in vivo.

### CS-SDFP/HA-Ag@MOF-PAL MN patches promoted skin repair

**Fig. 6:**
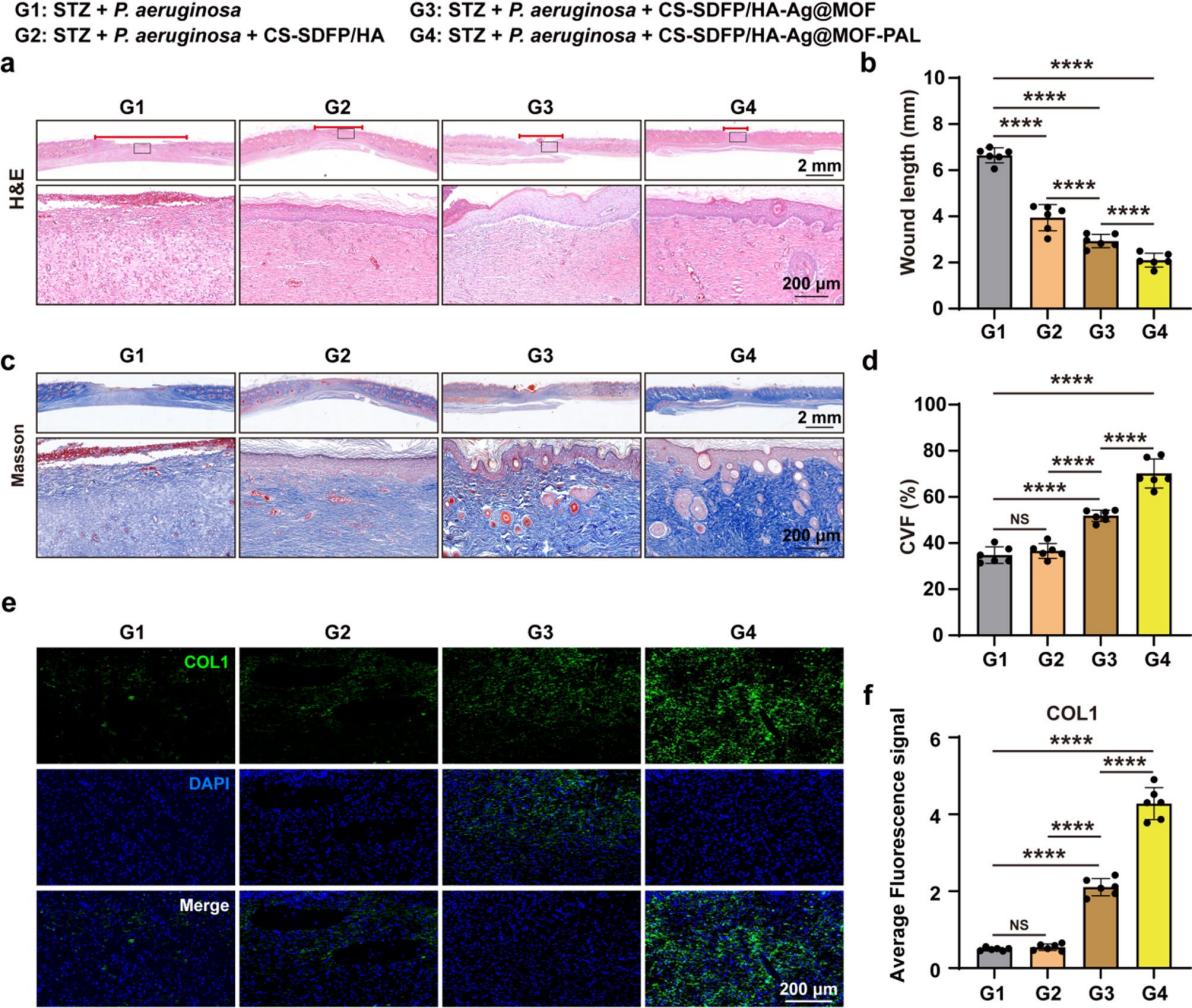
CS-SDFP/HA-Ag@MOF-PAL MN patches promoted skin repair. **a** Representative images of H&E staining. Scale bar = 2 mm and 200 μm. **b** Analysis of wound length. **c** Representative images of Masson trichrome staining. **d** The collagen volume fraction (CVF) for different treatments on day 18. **e** Representative immunofluorescence images of COL1α. Scale bar = 200 μm. and **f** its quantitative analysis. *p < 0.05, **p < 0.01, ***p < 0.001, ****p < 0.0001

To further validate the wound healing potential of CS-SDFP/HA-Ag@MOF-PAL MN patches, tissue regeneration at the wound area was evaluated following application of the MN patches. Hematoxylin and eosin (H&E) staining displayed near-complete wound closure in rats treated with CS-SDFP/HA-Ag@MOF-PAL MN patches on day 18 (Fig. 6a**)**. Compared with other groups, granulation tissue length was significantly shortened in the CS-SDFP/HA-Ag@MOF-PAL treated group (Fig. 6b). Collagen, a critical component of the skin and vital for wound healing and tissue remodeling [35], was assessed using Masson’s staining. On day 18, wounds exposed to CS-SDFP/HA-Ag@MOF-PAL MN patches exhibited enhanced collagen deposition (Fig. 6c), with a collagen volume fraction (CVF) reaching 70.18% ± 6.264%, which was markedly greater than that in the other groups (G1: 34.79% ± 3.558%; G2: 36.59% ± 3.197%; G3: 51.83% ± 2.497%) (Fig. 6d). Furthermore, immunofluorescence images revealed increased expression of COL1α protein following treatment with CS-SDFP/HA-Ag@MOF-PAL MN patches (Fig. 6e-f). These findings suggest that CS-SDFP/HA-Ag@MOF-PAL MN patches promote angiogenesis at the wound site. Consequently, CS-SDFP/HA-Ag@MOF-PAL MN patches effectively accelerated the healing of infected wounds in diabetic SD rats.

### The anti-inflammatory effects of CS-SDFP/HA-Ag@MOF-PAL MN patches

**Fig. 7:**
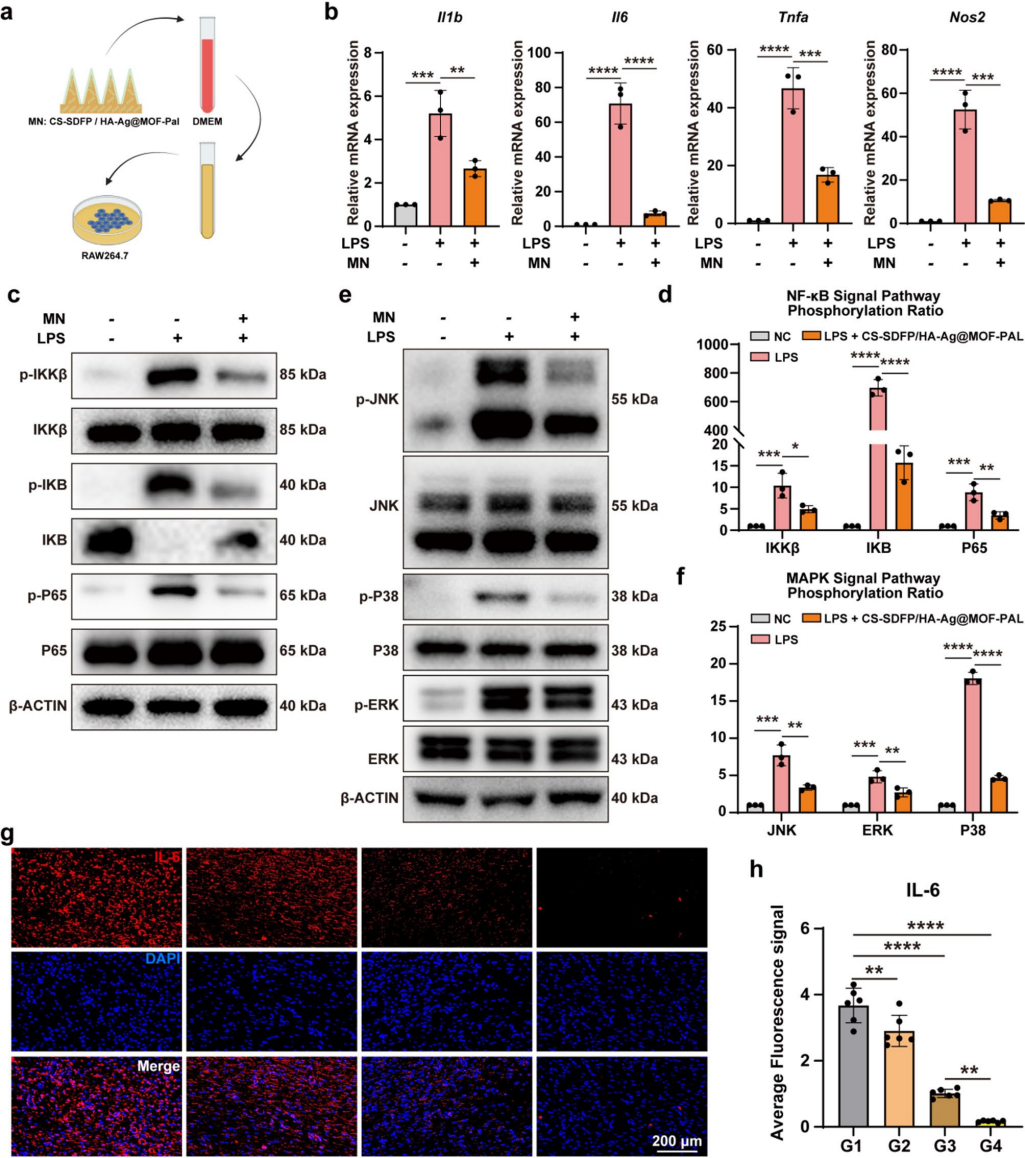
The anti-inflammatory effects of CS-SDFP/HA-Ag@MOF-PAL MN patches. **a** Scheme of the experiment *in vivro*. **b** Relative mRNA expression of inflammatory index in RAW264.7 after treatment with LPS (100 ng/ml) and CS-SDFP/HA-Ag@MOF-PAL MN patches. **c** Representative western blot of NF-κB signaling pathways in RAW264.7 after treatment with with LPS (100 ng/ml) and CS-SDFP/HA-Ag@MOF-PAL MN patches for 6 h. **d** The phosphorylation ratio of NF-κB signaling pathways. **e** Representative western blot of MAPK signaling pathways in RAW264.7 after treatment with with LPS (100 ng/ml) and CS-SDFP/HA-Ag@MOF-PAL MN patches for 6 h. **f** The phosphorylation ratio of MAPK signaling pathways. **g** Representative immunofluorescence images of IL-6. Scale bar = 200 μm. and **h** its quantitative analysis. *p < 0.05, **p < 0.01, ***p < 0.001, ****p < 0.0001

The CCK8 assay demonstrated that PAL exhibited no toxicity to HUVECs (Fig. S2a). After LPS (100 ng/ml) stimulation, significant upregulation was observed in the expression of *Il1b*, *Il6*, *Tnfa*, and *Inos*, but these changes were notably reversed following PAL administration (Fig. S2b). The MN patches were added into 5 ml DMEM/HG completed medium to make extracts of MN patches. To further assess the anti-inflammatory effects of the MN patches, extracts from CS-SDFP/HA-Ag@MOF-PAL MN patches were used to treat RAW 264.7 cells (Fig. 7a). Following treatment with MN extracts, LPS-induced hyperexpression of inflammatory markers such as *Il1b*, *Il6*, *Tnfa*, and *Nos2* was significantly inhibited (Fig. 7b). The nuclear factor kappa-B (NF-κB) and mitogen-activated protein kinase (MAPK) signaling cascades are both implicated in numerous chronic inflammatory disorders [36–38]. To investigate whether CS-SDFP/HA-Ag@MOF-PAL MN patches affected these pathways, Western blot analysis was performed. Administration of CS-SDFP/HA-Ag@MOF-PAL MN patches significantly decreased the phosphorylation levels of NF-κB (IKKα/β, P65, and IKBα) and MAPK (JNK, ERK, and P38) pathways, which were activated by LPS treatment (Fig. 7c–f). Immunohistochemical staining for IL-6 revealed that CS-SDFP/HA-Ag@MOF-PAL treatment resulted in a significant reduction of inflammation at the wound site, with a notable decrease in IL-6 secretion compared to the other groups (Fig. 7g, h). This finding underscores the potent anti-inflammatory effect of CS-SDFP/HA-Ag@MOF-PAL.

### CS-SDFP/HA-Ag@MOF-PAL recruit stem cells and promote angiogenesis

**Fig. 8:**
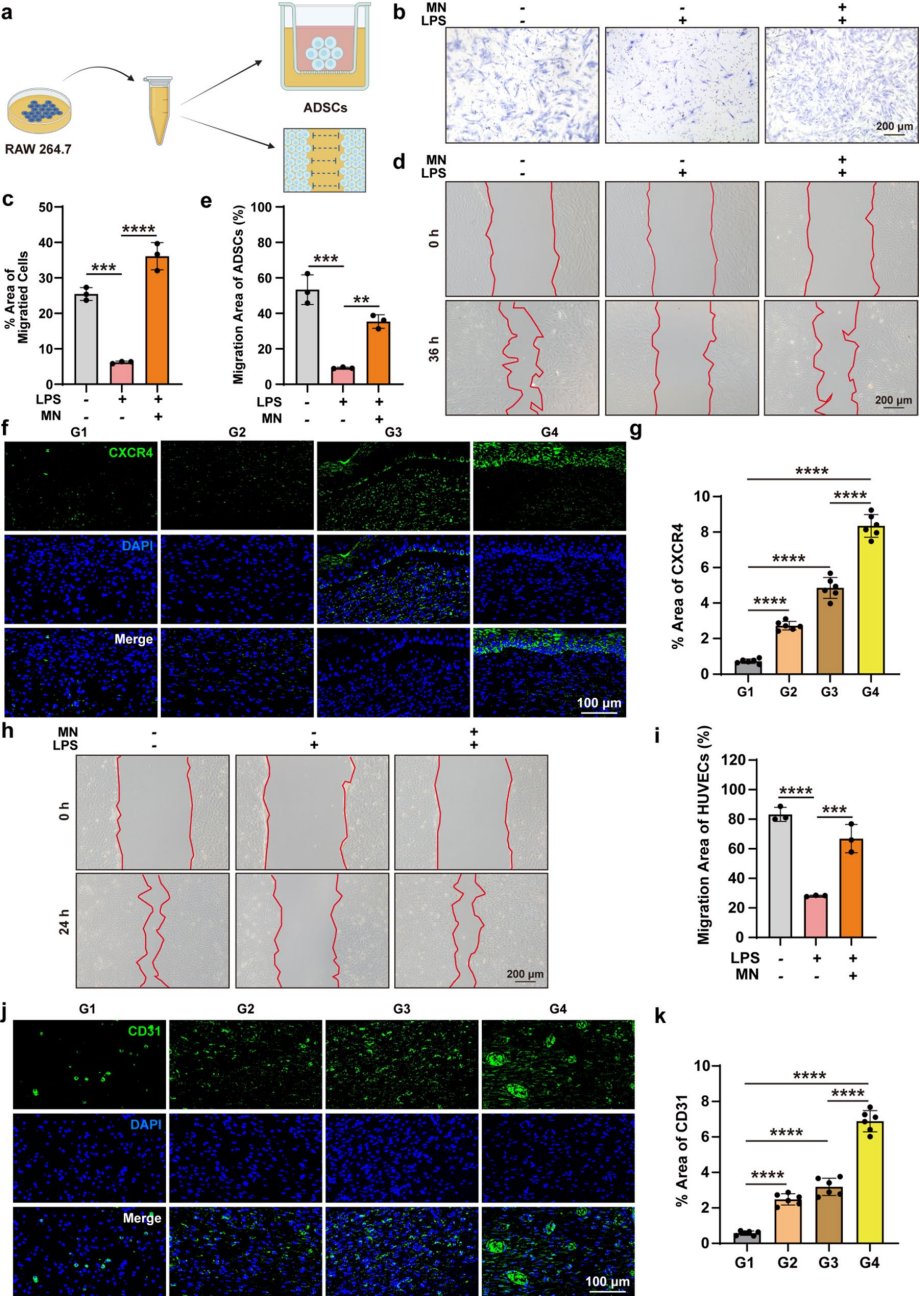
CS-SDFP/HA-Ag@MOF-PAL recruit stem cells and promote angiogenesis. **a** Scheme of the ADSCs treatment. **b** Bright-field images of migrated ADSCs after incubation with conditioned medium of RAW 264.7 cells and CS-SDFP/HA-Ag@MOF-PAL MN patches. Scale bar = 200 μm. **c** The quantification of (**b**). **d** Wound healing assay of the ADSCs after incubation with conditioned medium of RAW 264.7 cells and CS-SDFP/HA-Ag@MOF-PAL MN patches. Scale bar = 200 μm. **e** Quantitative analysis of the rate of wound closure at 36 h. **f** Representative immunofluorescence images of CXCR4. Scale bar = 100 μm. and (**g**) its quantitative analysis. **h** Wound healing assay of the HUVECs after incubation with conditioned medium of RAW 264.7 cells and CS-SDFP/HA-Ag@MOF-PAL MN patches. Scale bar = 200 μm. **i** Quantitative analysis of the rate of wound closure at 24 h. **j** Representative immunofluorescence images of CXCR4. Scale bar = 100 μm. and **k** its quantitative analysis

Adipose-derived stem cells (ADSCs), widely found in adipose tissue, are integral to a variety of therapeutic effects via paracrine mechanisms, including promoting angiogenesis, inhibiting apoptosis, and regulating immune responses [39]. The CCK8 assay demonstrated that SDFP had no toxicity to HUVECs (Fig. S3a) or ADSCs (Fig. S3b). Transwell assays (Fig. S3c-d) confirmed that SDFP concentrations accelerated ADSCs migration in vitro. The ADSCs underwent co-cultivation in RAW 264.7 cell-derived conditioned medium obtained from various stimulation conditions (Fig. 8a**)**. In the inflammatory environment, ADSC migration was inhibited. However, CS-SDFP/HA-Ag@MOF-PAL treatment significantly accelerated ADSC migration in vitro (Fig. 8b–e). C-X-C chemokine receptor type 4 (CXCR4), a receptor for SDF-1α, was highly expressed in ADSCs [40]. Immunofluorescent staining for CXCR4 revealed that among all the experimental groups, G4 had the highest proportion of ADSCs (8.35% ± 0.636%) (Fig. 8f, g). Scratch assays demonstrated that HUVECs migration was impaired in an inflammatory environment. However, CS-SDFP/HA-Ag@MOF-PAL therapy accelerated HUVECs migration in vitro (Fig. 8h-i). Immunofluorescent staining for CD31 indicated a significantly higher microvessel density (6.89% ± 0.6%) at the wound site in the CS-SDFP/HA-Ag@MOF-PAL group (Fig. 8j, k). These results suggest that CS-SDFP/HA-Ag@MOF-PAL MN patches effectively recruit stem cells and promote angiogenesis.

## Discussion

In this study, a multifunctional core-shell MN patch was engineered, exhibiting antibacterial and anti-inflammatory properties while promoting ADSC migration to enhance the healing of infected wounds. The core consists of HA-Ag@MOF-PAL, and the shell, composed of CS, encapsulates the SDFP. Upon insertion, the shell swells, while the core dissolves, releasing Ag@MOF-PAL. Ag@MOF demonstrated the ability to eradicate common wound-infecting bacteria, encompassing *S. aureus*, *E. coli*, and *P. aeruginosa*. In a diabetic rat model with *P. aeruginosa*-infected wounds, application of the CS-SDFP/HA-Ag@MOF-PAL MN patch markedly improved bacterial elimination, accelerated wound closure, mitigated inflammatory responses, and supported both tissue regeneration and angiogenesis versus control groups. This MN patches had no toxicity to the heart, liver, spleen, lungs and kidneys of rats. These findings highlight the considerable promise of this multifunctional MN patch in managing chronic wound infections.

Chronic inflammation in diabetic wounds, mediated by M1 macrophages, results in the sustained release of inflammatory cytokines, proteolytic enzymes, and reactive oxygen species (ROS), which in turn promote extracellular matrix (ECM) degradation [41]. Chronic inflammatory conditions, such as diabetic wounds [19, 42, 43], osteoarthritis [44], and neuroinflammation in spinal cord injury [45], are closely associated with the activation of the NF-κB and MAPK signaling pathways. In the context of diabetic wounds, the multifunctional MN patch releases PAL upon core dissolution, exerting potent anti-inflammatory effects. PAL suppressed the expression of inflammatory cytokines like IL-1β, IL-6, and TNFα, while treatment with CS-SDFP/HA-Ag@MOF-PAL markedly diminished the phosphorylation of critical proteins involved in the NF-κB and MAPK signaling pathways. These results demonstrate the anti-inflammatory efficacy of the MN patch, which accelerates wound healing.

Extensive tissue loss presents a major obstacle to the effective healing of infected diabetic wounds [46, 47]. In the presence of bacterial infection and elevated inflammation, cell proliferation is impaired, and apoptosis is exacerbated. Once Ag@MOF eliminates the bacteria and PAL attenuates the inflammatory response, the swollen CS shell encapsulating SDFP begins to perform its function. SDFP, a short chemotactic peptide fragment (sequence SKPVVLSYR) derived from SDF-1α, shares the same receptor activation domain as the full-length SDF-1α protein [48]. Compared to complete proteins, SDFP is a more cost-effective alternative with comparable efficacy in promoting stem cell migration [28]. Furthermore, substantial evidence supports that SDF-1α plays a key role in stimulating angiogenesis [49]. Following treatment with the multifunctional MN patch, the wound microenvironment was restored to a near-normal state. The swollen CS shell encapsulating SDFP attracted surrounding ADSCs, facilitated angiogenesis, and contributed to the repair of the tissue defect.

In summary, the CS-SDFP/HA-Ag@MOF-PAL MN patch has shown promising efficacy in promoting wound healing. It effectively combines antibacterial, anti-inflammatory, ADSC-recruitment, and angiogenic activities. In the animal experiments of this study, after 18 days of observation, CS-SDFP/HA-Ag@MOF-PAL MN patch did not exhibit any biological toxicity. Next, we will further explore the long-term biocompatibility concerns of CS-SDFP/HA-Ag@MOF-PAL MN patch. The CS-SDFP/HA-Ag@MOF-PAL MN patch holds potential as a novel strategy in the future treatment of infected diabetic wounds.

## Materials and methods

### Bacteria and cell lines

The *E. coli*, *S. aureus* and *P. aeruginosa* bacteria were procured from the American Type Culture Collection (ATCC, USA). The *E. coli*, and *P. aeruginosa* bacteria were cultivated in lysogeny broth (LB), and the *S. aureus* was propagated in Tryptic Soy Broth (TSB) nutrient broth.

Human umbilical vein endothelial cells (HUVECs) (RRID: CVCL_0493) and RAW264.7 cells (RRID: CVCL_E5ZU) were procured from Haixing Biosciences in December 2023. After identification, none of the cell lines were contaminated or misidentified. HUVECs were cultured in complete RPMI-1640 (Boster, China). RAW264.7 cells were cultured in complete DMEM/HG (Boster, China). To harvest the primary ADSCs, groin adipose was dissected from 6-week-old Sprague-Dawley (SD) rats. Adipose tissue was minced and enzymatically digested using 0.25% type II collagenase prepared in Dulbecco’s Modified Eagle Medium (DMEM)/F12 (Hyclone, USA). The prepared cell suspension underwent cultivation in a complete medium comprising 10% FBS (Gibco, USA) and 1% penicillin-streptomycin (Boster, China) under standard incubation conditions of 5% CO₂ at 37 ℃.

### Synthesis of Ag@MOF

Based on established research, Ag@MOF [Ag5(PYDC)2(OH)] preparation involved a hydrothermal synthesis approach [50]. In the procedure, silver nitrate (AgNO₃) (MACKLIN, China) (0.30 g) and pyridine-3,5-dicarboxylic acid (H₂PYDC) (Aladdin, China) (0.14 g) underwent dissolution in deionized water (20 ml). Following thorough agitation to achieve complete dissolution, the solution was transferred into a 50 ml Teflon-lined stainless steel autoclave for thermal treatment at 120 °C lasting 24 h. The obtained colorless crystalline material underwent purification through multiple centrifugation cycles using deionized water. The final Ag@MOF product was subjected to vacuum drying at 80 °C for 18 h.

### Fabrication of the CS-SDFP/HA-Ag@MOF-PAL MN patches

The CS (2 g, Aladdin, China, < 200 mPa.s) was dissolved in 100 mL of 1% hydrochloric acid. The resulting solution was dialyzed against double-distilled water (ddH₂O) utilizing a dialysis membrane with a molecular weight cut-off (MWCO) of 3500 Da for 2 days to eliminate residual hydrochloric acid. The CS solution was subsequently concentrated to 20 mL at 60 °C. Thereafter, 20 mg of SDFP was incorporated into the CS solution and agitated under cold conditions.

A mixture of 100 mg of Ag@MOF and 100 mg of PAL was prepared by dissolving both components in 50 mL ddH_2_O, followed by stirring on ice for 24 h. The Ag@MOF-PAL composite was subsequently isolated through centrifugation at 10,000 rpm for 10 min, succeeded by separation from the supernatant and freeze-drying of the resulting material. The drug concentration in the supernatant was subsequently measured. For the HA solution, 16 g of HA and 16 g of sucrose were dissolved in 100 mL ddH_2_O.

The CS-SDFP mixture (50 µl) was deposited onto a PDMS mold surface, subsequently positioned on a vacuum chuck for 30 min at -2.2 MPa vacuum pressure. Following the CS-SDFP desiccation, 100 µL of Ag@MOF-PAL was introduced onto the PDMS mold surface (covering the CS-SDFP layer). The mold underwent vacuum treatment for 2 h. Subsequently, the mold remained in a desiccator at ambient temperature for 48 h to achieve complete dehydration. The final step involved carefully extracting the MN patch from the mold, succeeded by storage in a desiccator until required.

### Hemolysis experiment

To evaluate the biosafety of CS-SDFP/HA-Ag@MOF-PAL MN patches, 1 mL of mouse blood was drawn and transferred into a tube comprising sodium heparin. The sample was rinsed five times with 3 mL of saline, succeeded by gentle shaking and centrifugation at 1500 rpm for 10 min until a clear supernatant was observed. Red blood cells were resuspended in saline and aliquoted into five separate tubes, corresponding to the following groups: H₂O, Normal Saline (NS), CS-SDFP/HA, CS-SDFP/HA-Ag@MOF, and CS-SDFP/HA-Ag@MOF-PAL. Extracts from the MN patches of each group were introduced into the red blood cell suspensions and incubated overnight at 4 °C. Following centrifugation at 1500 rpm for 10 min, the supernatant was retrieved, and the absorbance at 540 nm was recorded to evaluate the hemolysis rate.

### Cell counting Kit-8 (CCK-8) assay

Cell Counting Kit-8 assay (CCK8, Boster, China) was utilized to evaluate the effects of MN patch, PAL, and SDFP on HUVECs viability. HUVECs (8000 cells/well) were placed in 96-well plates, adhered for 24 h, and subsequently treated as described below. They were administered diverse concentrations of the PAL (100, 150, 200, 250 µM), SDFP (0.5, 1, 1.5, 2 µg/ml), or different MN patches. After a 24 h exposure phase, 100 µl of CCK8 solution was dispensed into individual wells, succeeded by a 1 h incubation period. Subsequently, the OD measurements were procured at 450 nm wavelength utilizing a microplate reader.

### Antibacterial effect of CS-SDFP/HA-Ag@MOF-PAL MN patch

To assess bacterial viability following treatment with various MN patches, *S. aureus*, *E. coli*, and *P. aeruginosa* were stained using the LIVE/DEAD Bacterial Staining Kit with DMAO & PI (Beyotime, China). First, different MN patches (CS-SDFP/HA, CS-SDFP/HA-Ag@MOF, CS-SDFP/HA-Ag@MOF-PAL) were cultured in PBS for 24 h. The PBS solutions containing the MN patches (MN-PBS) were used in subsequent experiments. The bacterial cultures were separated into four identical portions, with MN-PBS introduction to the bacterial specimens, pursued by a 4-hour cultivation at 37 °C. Following centrifugation (5000 rpm, 5 min) and removal of supernatant fluid, the bacterial pellet was resuspended in 100 µL of DMAO & PI staining solution. The specimens underwent dark incubation for 15 min, succeeded by three PBS washing cycles. Subsequently, 100 µL of PBS was incorporated to generate a uniform bacterial suspension suitable for fluorescence microscopy at 60× magnification. The ImageJ software was used to measure the images. Bacterial mortality was calculated as the ratio of dead to total bacteria:

$$\begin{aligned} {Bacterial \; mortality} = {(Dead \; bacteria \; count/} \\{Total\; bacteria\;count)} \times 100 \% . \end{aligned}$$

### Bacterial morphology

To observe bacterial morphology following treatment with different MN patches, FESEM was employed. *S. aureus*, *E. coli*, and *P. aeruginosa* were cultured in LB liquid medium for 24 h, then harvested by centrifugation at 5000 rpm for 5 min. MN-PBS was added to the bacterial pellets, followed by incubation at 37 °C for 4 h. The bacterial cells were subsequently recollected by centrifugation under the same conditions. Afterward, the samples underwent three washes with PBS, succeeded by cell fixation in 2.5% glutaraldehyde solution during overnight storage at 4 °C. Another centrifugation at 5000 rpm for 5 min was performed, after which dehydration was conducted stepwise. The bacteria were sequentially immersed in ethanol solutions of 30%, 50%, 70%, 90%, and 100%, each for 15 min. Finally, the samples were resuspended in absolute ethanol, and bacterial morphology was observed on silicon slides using FESEM.

### Determination of the Inhibition zone

To evaluate the antimicrobial efficacy, 50 µL bacterial suspensions of *S. aureus*, *E. coli*, and *P. aeruginosa* (concentration: 10^8^ CFU/mL) were spread onto LB solid medium plates. Various MN patches (CS-SDFP/HA, CS-SDFP/HA-Ag@MOF, CS-SDFP/HA-Ag@MOF-PAL) were positioned centrally on the LB solid medium, succeeded by incubation at 37 °C for 24 h. The diameters of the inhibition zones were ascertained, and photos were taken using an Apple iPhone 15 Pro Max. Each experimental group was performed in triplicate.

### Biofilm clearance experiment

*S. aureus* underwent cultivation in LB medium comprising 3% sucrose, while *E. coli* and *P. aeruginosa* were grown in standard LB medium for 48 h to induce biofilm formation. The resulting biofilms were then incubated with various MN patches in fresh LB medium for 12 h. Post-treatment, the supernatant was carefully extracted, and the biofilms underwent fixation with methanol for 30 min. The samples were subjected to staining using 0.1% crystal violet solution for 30 min. Following image acquisition, the stained biofilm underwent solubilization in 33% glacial acetic acid, and measurements of absorbance at 595 nm were performed using a UV spectrophotometer to determine the biofilm clearance rate.

### Wound healing test

The work has been reported in line with the ARRIVE guidelines 2.0. To further evaluate the effectiveness of CS-SDFP/HA-Ag@MOF-PAL MN patches in promoting healing of infected diabetic wounds, a rat diabetic wound model was established. All animal experiments were sanctioned by the Experimental Animal Welfare Ethics Committee of Zhongnan Hospital, Wuhan University (ZN 2025063). Male SD rats (8 weeks old) were obtained from Hubei Biont Biological Technology Co., Ltd. Initially, 10 mg of streptozotocin (STZ) (Macklin, China) was dissolved in 1 mL of sodium citrate buffer (Phygene Scientific, China) and filtered through a 0.22 μm filter. Prior to the experimental procedure, the blood glucose levels of SD rats were determined. Next, the rats were intraperitoneally administered 60 mg/kg/day of STZ solution for 5 days. After one week, blood glucose levels were measured again. After anesthesia was administered using pentobarbital sodium, a circular wound measuring 1.5 cm in diameter was created on the dorsal area of each rat. A 100 µL suspension of *P. aeruginosa* (10⁸ CFU/mL) was applied to the wound for 2 days to develop a diabetic wound infection model. Subsequently, the rats were divided into 4 groups randomly and received treatment with various MN patches, each comprising six subjects (*n* = 6).

- Group 1 (G1) received no treatment, *n* = 6;
- G2 received CS-SDFP/HA, *n* = 6;
- G3 received CS-SDFP/HA-Ag@MOF, *n* = 6;
- G4 received CS-SDFP/HA-Ag@MOF-PAL, *n* = 6.

Photographic records of the wounds were obtained on days − 2, 0, 3, 7, 10, 14, and 18 subsequent to the treatment implementation. The wound-remaining ratio was computed using the following formula:

$$ \begin{aligned} {Wound-remaining\;ratio\;(\%)} = {(Actual\;wound\;area/}\\{\text{Original wound area}})\times 100\% . \end{aligned}$$

Additionally, *P. aeruginosa* obtained from the lesions was cultivated on an LB solid medium to monitor microbial proliferation and assess the antimicrobial efficacy across diverse treatment groups.

### Histological analysis

The skin around the wounds was cut off after the rats were sacrificed by CO_2_. The skin was spread flat on the slide and submerged in 4% paraformaldehyde (PFA) for 2 days. Subsequently, the samples underwent paraffin embedding and were precisely sectioned into tissue slices with a thickness of 5 μm for histological examination. ImageJ software was employed to precisely quantify the epidermal thickness, the volume fraction of collagen, and the coverage of neovascularization within the wound area. Furthermore, immunofluorescence (IF) was utilized to measure the expression levels of the relevant proteins.

### RNA extraction and real-time PCR

After exposure to PAL or different MN patches, RAW 264.7 cells underwent triple PBS washing. Cell lysis was performed utilizing RNA Isolator Total RNA Extraction Reagent (Vazyme, China). The extracted mRNA (1 µg) was reverse-transcribed to cDNA through HiScript II Q RT SuperMix (Vazyme, China). The primer sequences can be found in Supplementary Table 1. For cDNA detection, Taq Pro Universal SYBR qPCR Master Mix (Vazyme, China, Q712-02) was applied. Fluorescence monitoring during amplification was conducted via a real-time PCR detection system (Bio-Rad, USA). The 2^–ΔΔCt^ method enabled calculation of target mRNA relative expression levels.

### Western blot

RAW 264.7 cells underwent exposure to lipopolysaccharide (LPS) at 100 ng/mL concentration, either alone or combined with CS-SDFP/HA-Ag@MOF-PAL MN patches across a 6-hour period. Subsequently, cellular material was lysed utilizing RIPA buffer (Boster, China) containing 1% protease and phosphatase inhibitors (GServicebio, China). The extracted lysates underwent centrifugation at 12,000 rpm maintained at 4 °C for 20 min. The obtained supernatants were combined with loading buffer (Servicebio, China) in a 1:4 proportion and subjected to heating at 97 °C for 10 min. Identical protein quantities were subjected to SDS-PAGE gel electrophoresis for separation. Subsequently, the protein components were transferred onto polyvinylidene fluoride (PVDF) membranes (Millipore, USA). These membranes underwent blocking in TBST containing 5% bovine serum albumin (BioFroxx, Germany) for 1 h. Primary antibody application occurred at 4 °C overnight (Supplementary Table 2), followed by secondary antibody treatment for 1 h. Signal detection was accomplished using an enhanced chemiluminescence kit (Vazyme, China) and documented via ChemiDoc XRS System (Bio-Rad, USA). Protein levels were analyzed through ImageJ software.

### Transwell

In this assay, a 24-well transwell plate measuring 6.5 mm, equipped with an insert featuring an 8.0 μm pore polycarbonate membrane (Corning, USA), was employed. DMEM/F12 (10% FBS) containing CS-SDFP/HA-Ag@MOF-PAL MN patches or different concentrations of SDFP was added to the lower chamber. The ADSCs were seeded at a density of 2.0 × 10⁴ cells per well into the upper chamber filled with serum-free medium. After 24 h of incubation, cotton swabs were used to scrape off the non-migrating cells on the upper side of the chamber. The migrating cells on the lower side of the membrane were fixed with 4% PFA and stained with 0.1% crystal violet dye. The cells that had been stained were enumerated and subjected to analysis with the use of ImageJ software.

### Statistical analysis

All data are denoted in the form of mean ± standard deviation (SD). For statistical analysis, GraphPad Prism 9.0 software was utilized. To ensure the reliability of the data analysis, at least three biological replicates were employed. When comparing multiple samples, one-way analysis of variance (ANOVA) was conducted. For pairwise comparisons of group means, Student’s t-test was applied. The asterisks (*, **, ***, ****) are utilized to denote p values of less than 0.05, 0.01, 0.001, and 0.0001, respectively. Statistical significance was determined by a p value less than 0.05.

## Supplementary Information

Supplementary material 1.

## Data Availability

Data are available from the corresponding author upon reasonable request.
