# Supplementary material for: A core-shell multifunctional microneedle patch accelerates infected diabetic wound healing
Source: Stem Cell Res Ther. 2025 Oct 31;16:601. doi: 10.1186/s13287-025-04738-z (PMC12577055; doi:10.1186/s13287-025-04738-z)
Supplement: Supplementary file 1 — Supplementary material 1. [file 13287_2025_4738_MOESM1_ESM.docx]

**Supplementary Information**

**Supplementary Table 1.** primer sequences for RT-PCR.

| Gene |  | Primer sequence (5′–3′) |
| --- | --- | --- |
| *Actb* | F | GTGACGTTGACATCCGTAAAGA |
|  | R | GCCGGACTCATCGTACTCC |
| *Il1b* | F | CTTTTTTGTTGTTCATCTC |
|  | R | AGGCAGTATCACTCATTGT |
| *Il6* | F | GGCTTTGTCTTTCTTGTTA |
|  | R | TCTTGGGACTGATGCTGGT |
| *Tnfa* | F | TGGTGGTTTGTGAGTGTGA |
|  | R | TACTGAACTTCGGGGTGAT |
| *Nos2* | F | CTCTCATCCAGAACCTCCA |
|  | R | ATCCCGAAACGCTTCACTT |

**Supplementary Table 2.** Antibody information.

| Antibody | Brand | Application and Dilution |
| --- | --- | --- |
| p-IKKβ | CST, 2697T | 1:1000 |
| IKKβ | Proteintech, 15649-1-AP | 1:600 |
| p-IKBα | Proteintech, 82349-1-RR | 1:2000 |
| IKBα | Proteintech, 10268-1-AP | 1:5000 |
| p-P65 | Proteintech, 82335-1-RR | 1:3000 |
| P65 | Proteintech, 10745-1-AP | 1:3000 |
| p-JNK | Proteintech, 80024-1-RR | 1:2000 |
| JNK | Proteintech, 51153-1-AP | 1:1000 |
| p-P38 | Proteintech, 28796-1-AP | 1:2000 |
| P38 | Proteintech, 14064-1-AP | 1:3000 |
| p-ERK | Proteintech, 28733-1-AP | 1:3000 |
| ERK | Proteintech, 11257-1-AP | 1:3000 |
| β-ACTIN | Proteintech, 20536-1-AP | 1:5000 |


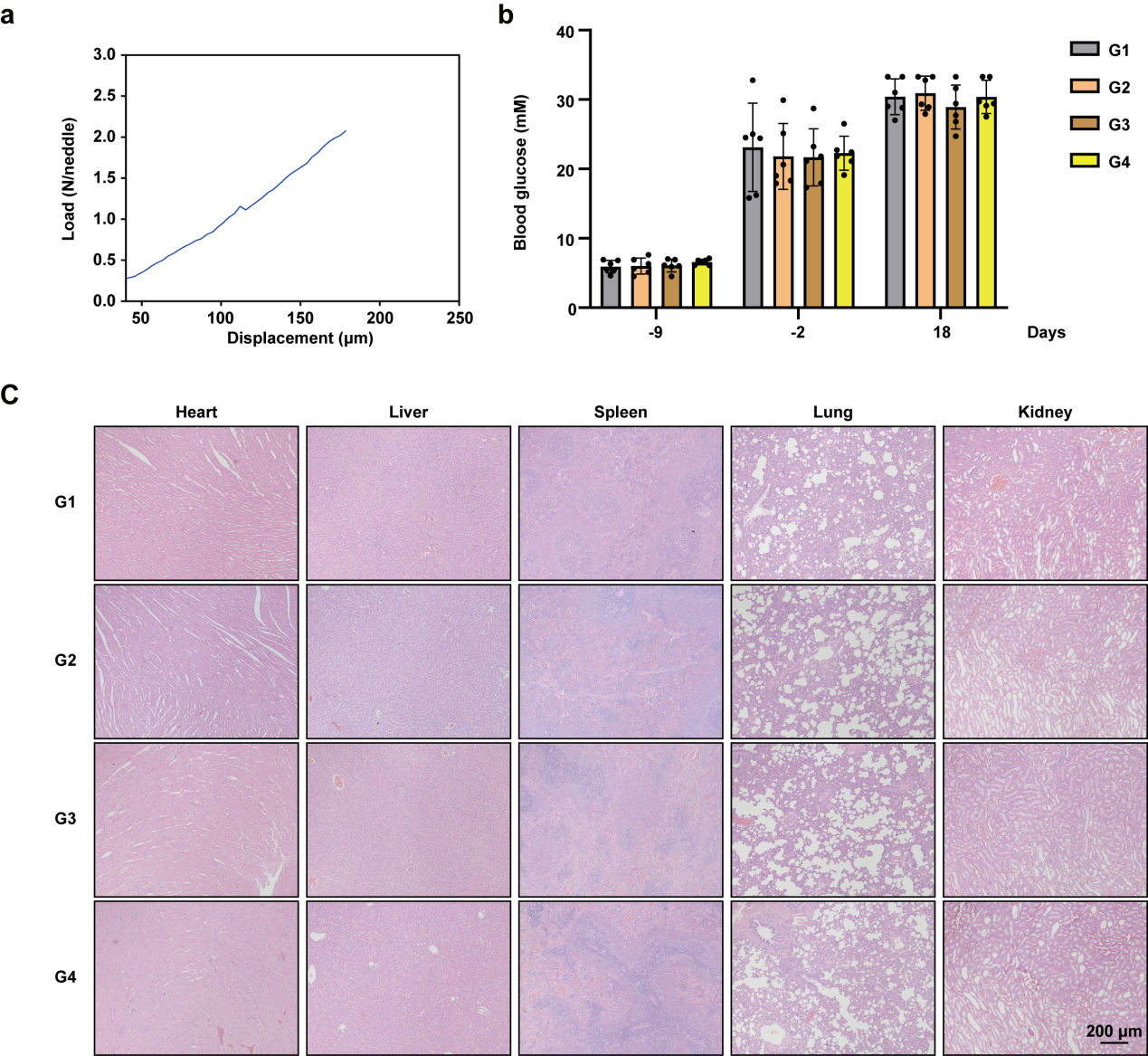


**Fig. S1** (a) Force-displacement curves of the CS-SDFP/HA-Ag@MOF-PAL MN patches. (b) The blood glucose of rats. (c) The H&E staining of Heart, liver, spleen, lung and kidney in rats after different MN treatment.


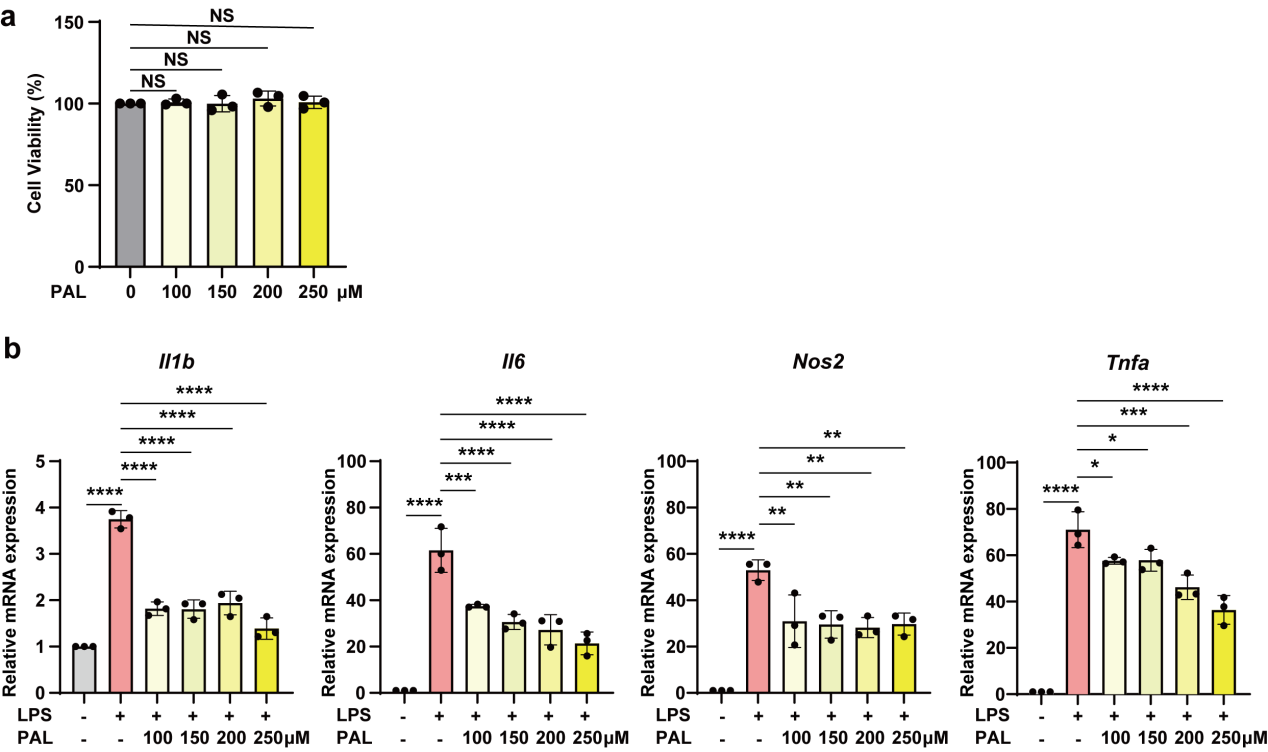


**Fig. S2** (a) Cell viability of HUVECs treated with PAL (0, 100, 150, 200, 250 μM). (b) Relative mRNA expression of inflammatory index in RAW264.7 after treatment with LPS (100 ng/ml) and different PAL concentration (0, 100, 150, 200, 250 μM).


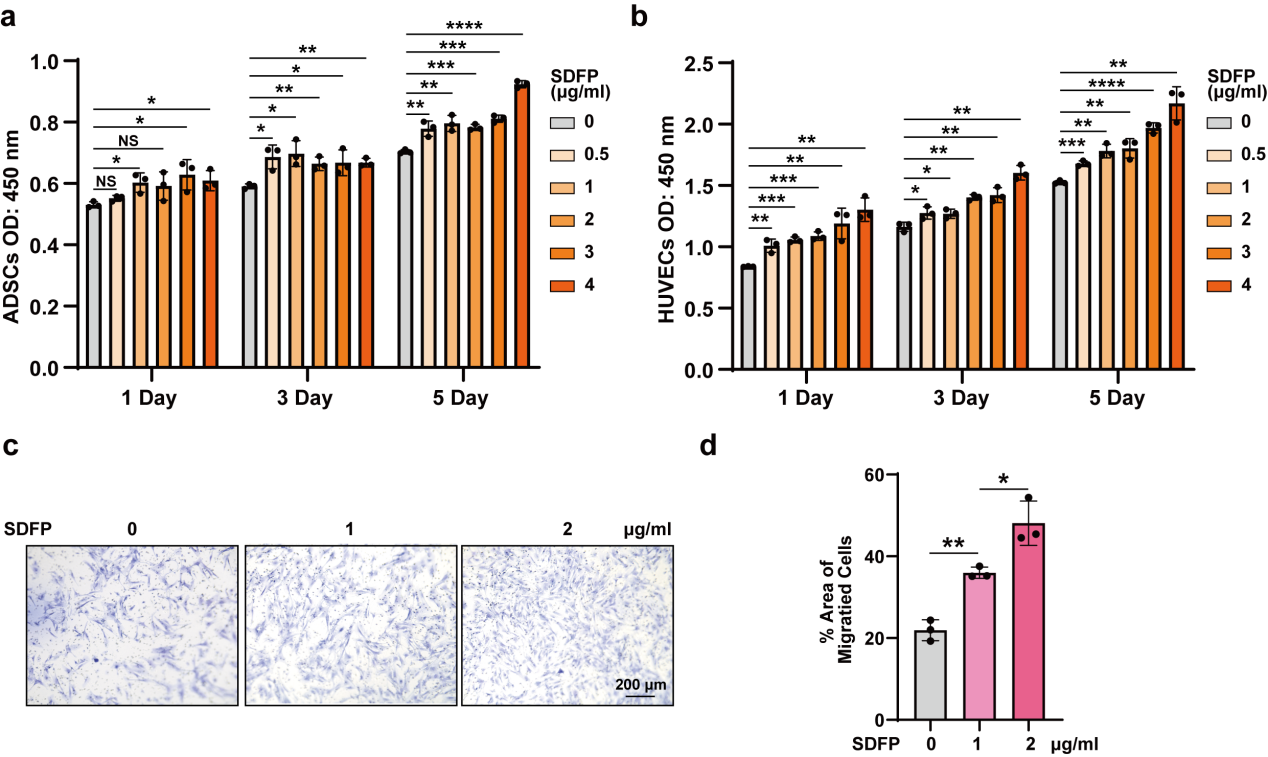


**Fig. S3** (a) Cell viability of HUVECs treated with SDFP (0, 0.5, 1, 2, 3, 4μg/mL). (b) Cell viability of ADSCs treated with SDFP (0, 0.5, 1, 2, 3, 4μg/mL). (c) Bright-field images of migrated ADSCs after incubation with different concentrations of SDFP. (d) The quantification of (c).


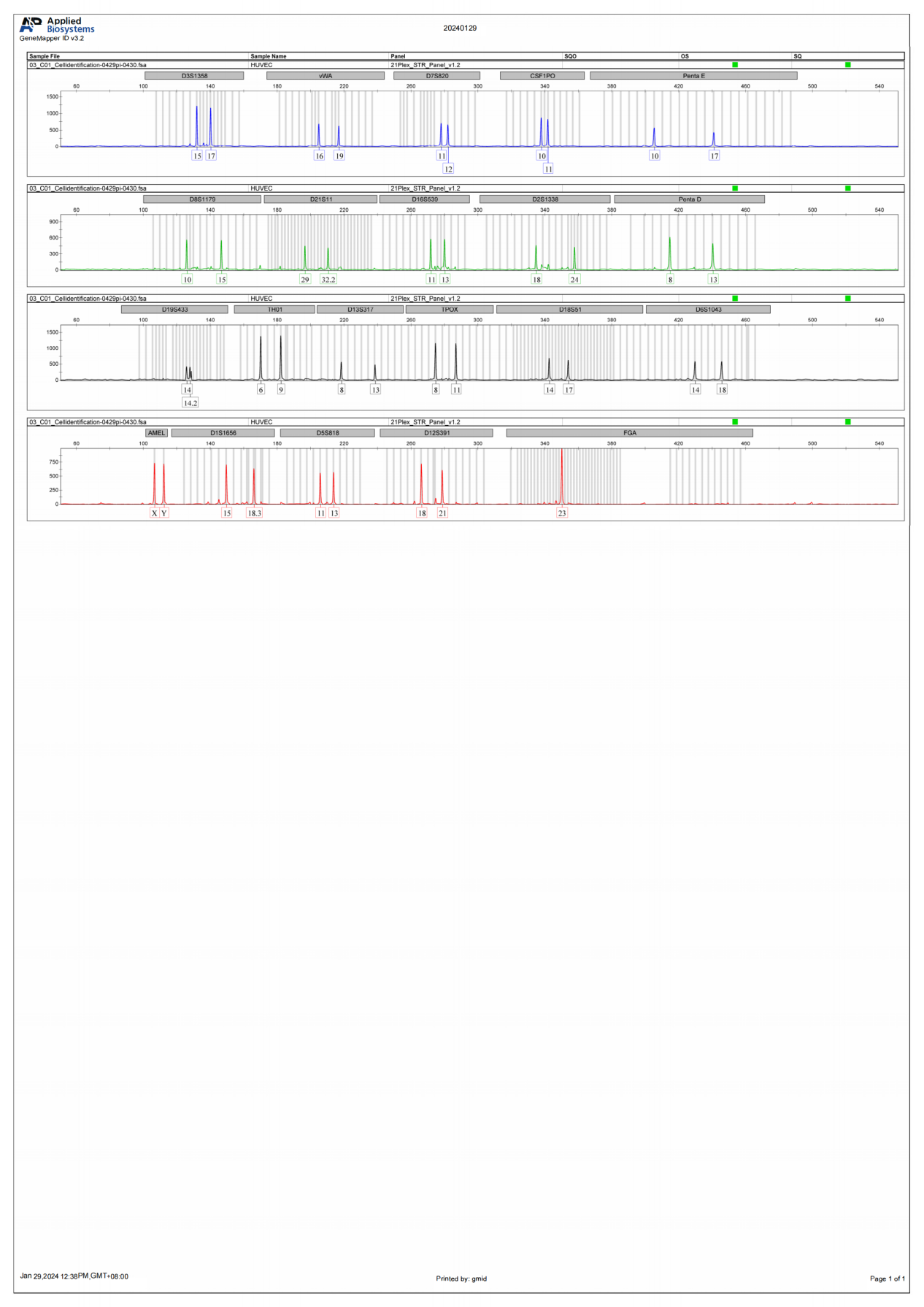


**Short Tandem Repeat (STR) of HUVECs supplied by Shanghai Fuheng Biological Technology Co., LT.**


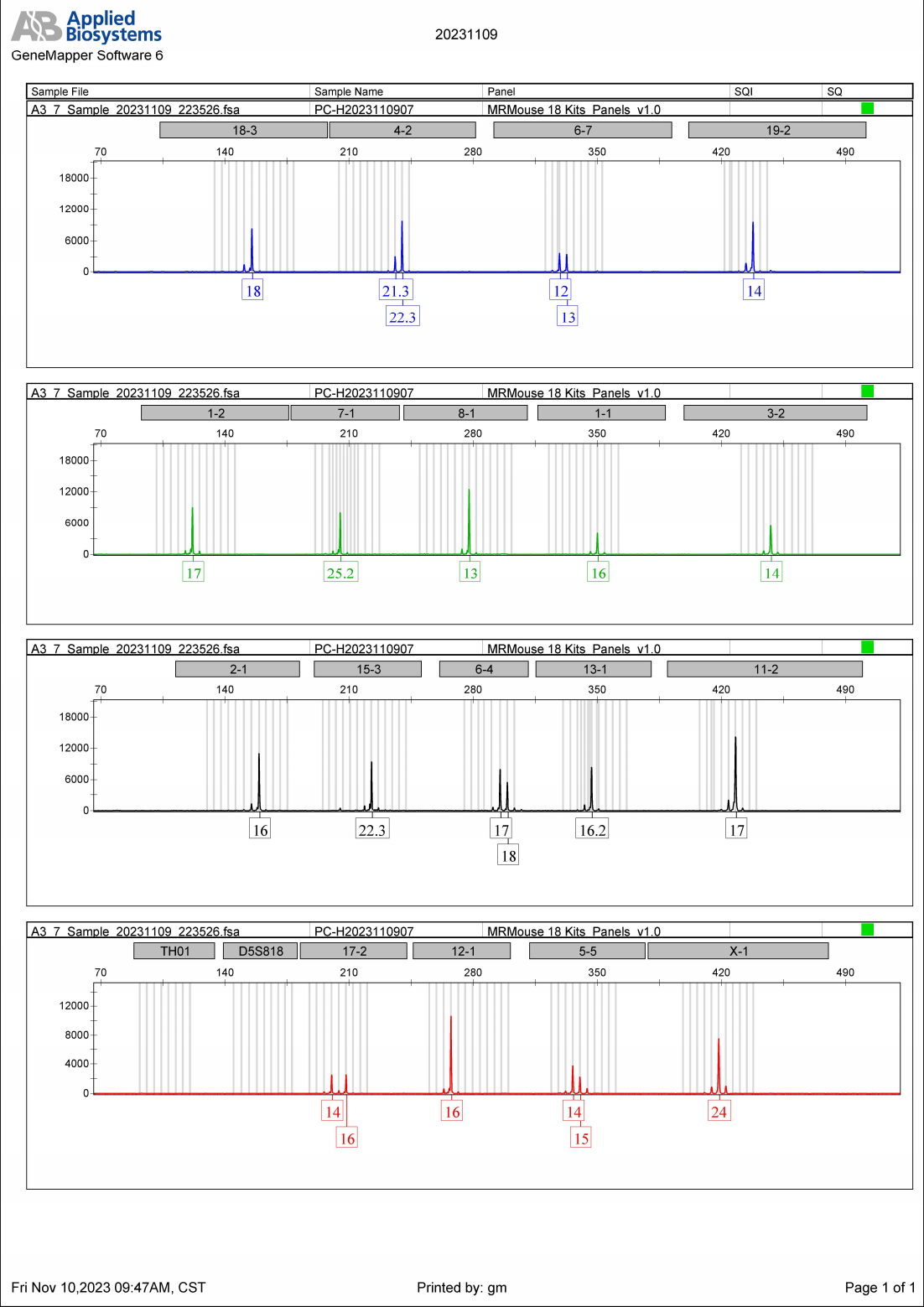


**STR of RAW 264.7 (PC-H2023110907) supplied by Haixing Biosciences.**
